# Inquiry, with Remarks

**Published:** 1875-06

**Authors:** 


———, GEORGIA, June 3, 1875.

*Dr. T. S. Powell:*

SIR—I am informed that there is a Medical Examining Board in the State of Georgia, and that you are a member of the same. The duty of this Board, as I understand, is to keep down quackery and imposture in the medical profession and among druggists. Yet in my own community, and in other places of my knowledge, quackery flourishes in all its phases. Druggists even prescribe as practitioners, and quack doctors and ignoramuses feast and fatten upon the ignorance, superstition, and credulity of the people, while the educated, honorable physician is, in many instances, passed by to starve, or compelled to leave the profession in disgust. How is this? What are the duties of your Board? How are these evils to be remedied? J. C. C.

In reply to the above communication, we desire to inform our correspondent that we are a member of the aforesaid Board, which has been in existence in this State since 1824.

This Board, during its long existence, has done a great deal of good in the interest of the medical profession, and in protecting the people from imposture by quacks. A law of the State, until recently, required physicians who practiced upon diploma to exhibit them to the Examining Board, and to secure from it license to practice. Such license were recorded in the county clerk's office. While this was the supervising duty of the Board, still it was not empowered under the law to act as prosecutor in cases where this provision was violated. As the law now stands, physicians who have diplomas are not required to exhibit them to the Board, or to obtain from it an additional license; but if men practice medicine who have no diploma from a regularly chartered medical college, they are required first to obtain a license from the Board representing the *system* under which they desire to practice—that is, the Allopathic Board, the Reform, and the Eclectic Board, or they must have a diploma from a regularly instituted Homeopathic college, if they wish to practice on that system. But all who practice homeopathy without such regular diploma, and all of those who practice under the various unauthorized "isms" under which quackery and charlatanism hides its deformity, and missleads and preys upon the unsuspecting public, are subject to the penalties of the law, viz: They shall be liable to indictment, and on conviction shall be fined, not exceeding five hundred dollars for the first offense, and, for the second, shall be imprisoned not more than two months—one-half of the fine to the informer, the other to the educational fund of the county.

Our correspondent will consequently see that the law is purely, in some respects, to protect legal practitioners and the public against quacks and impostors, as any citizen can inform the grand jury of his respective county any one whom he may have reason to believe is a violator of the low; whereupon, it becomes the duty of the grand jury to make the proper investigation, and to proceed against the offender according to the facts elicited. It should be remembered, too, that no Board of any of the aforementioned schools of medicine is authorized to grant license to practice under any other system than that represented by the Board granting said license. Persons practicing under license from the Allopathic Board can not practice medicine outside of that school, under the penalty of revokement of his license by the Board, upon sufficient evidence being made to it as to the offense.

We hold that, under the law, any physician who practices outside of the system from whose Borsd he holds license, is an offender against the spirit of the law, and should and can be indicted and punished according to the law. It is a solemn duty of the profession, and of every good citizen in each county, to take cognizance of this matter, and to see to it that offenders are promptly and effectually punished.

We hold, furthermore, that the spirit of the law makes it obligatory upon every practitioner to practice strictly in accordance with the medical system under which he hold his diploma, whenever and wherever he claims the right to practice under the shelter of said diploma, and we maintain that he becomes a violator of the law whenever he engages in practice which his Alma Mater would disclaim as quackery and foreign to its teachings and subversive of its doctrines. This mal-practice and quackery exists in the State, doubtless, but, as before said, the Examining Board have no prosecuting jurisdiction in such cases, as an official body. It, therefore, rests with the people themselves, and their grand juries, who alone have power to rid the community of these nuisances, and prevent the violation of the law for the protection of the public health.

